# Hot topics in biodiversity and climate change research

**DOI:** 10.12688/f1000research.6508.1

**Published:** 2015-09-30

**Authors:** Barry W. Brook, Damien A. Fordham

**Affiliations:** 1 School of Biological Sciences, Private Bag 55, University of Tasmania, Hobart, 7001, Australia; 2 The Environment Institute and School of Earth and Environmental Sciences, University of Adelaide, Adelaide, SA, 5005, Australia

**Keywords:** biodiversity, climate change, global change, conservation

## Abstract

With scientific and societal interest in biodiversity impacts of climate change growing enormously over the last decade, we analysed directions and biases in the recent most highly cited data papers in this field of research (from 2012 to 2014). The majority of this work relied on leveraging large databases of already collected historical information (but not paleo- or genetic data), and coupled these to new methodologies for making forward projections of shifts in species’ geographical ranges, with a focus on temperate and montane plants. A consistent finding was that the pace of climate-driven habitat change, along with increased frequency of extreme events, is outpacing the capacity of species or ecological communities to respond and adapt.

## Introduction

It is now halfway through the second decade of the 21 ^st^ century, and climate change impact has emerged as a “hot topic” in biodiversity research. In the early decades of the discipline of conservation biology (1970s and 1980s), effort was focused on studying and mitigating the four principal drivers of extinction risk since the turn of the 16 ^th^ century, colourfully framed by Diamond ^1^ as the “evil quartet”: habitat destruction, overhunting (or overexploitation of resources), introduced species, and chains of extinctions (including trophic cascades and co-extinctions). Recent work has also emphasised the importance of synergies among drivers of endangerment ^2^. But the momentum to understand how other aspects of global change (such as a disrupted climate system and pollution) add to, and reinforce, these threats has built since the Intergovernmental Panel on Climate Change reports ^3^ of 2001 and 2007 and the Millennium Ecosystem Assessment ^4^ in 2005.

**Figure 1.**
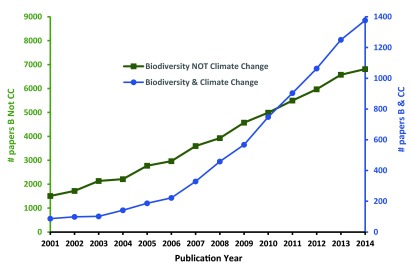
Relative growth of refereed studies on climate change and biodiversity, compared to non-climate-related biodiversity research. Number of refereed papers listed in the *Web of Science* database that were published between 2001 and 2014 on the specific topic “biodiversity AND (climate change)” (blue line, secondary y-axis) compared to the more general search term “biodiversity NOT (climate change)”.

Scientific studies on the effects of climate change on biodiversity have proliferated in recent decades. A *Web of Science* ( webofscience.com) query on the term “biodiversity AND (climate change)”, covering the 14 complete years of the 21 ^st^ century, shows the peer-reviewed literature matching this search term has grown from just 87 papers in 2001 to 1,377 in 2014. Figure 1 illustrates that recent scientific interest in climate change-related aspects of biodiversity research has outpaced—in relative terms—the baseline trend of interest in other areas of biodiversity research (i.e., matching the query “biodiversity NOT (climate change)”), with climate-related research rising from 5.5% of biodiversity papers in 2001 to 16.8% in 2014.

Interest in this field of research seems to have been driven by a number of concerns. First, there is an increasing societal and scientific consensus on the need to measure, predict (and, ultimately, mitigate) the impact of anthropogenic climate change ^5^, linked to the rise of industrial fossil-fuel combustion and land-use change ^6^. Biodiversity loss and ecosystem transformations, in particular, have been highlighted as possibly being amongst the most sensitive of Earth’s systems to global change ^7, 8^. Second, there is increasing attention given to quantifying the reinforcing (or occasionally stabilising) feedbacks between climate change and other impacts of human development, such as agricultural activities and land clearing, invasive species, exploitation of natural resources, and biotic interactions ^2, 9^. Third, there has been a trend towards increased accessibility of climate change data and predictions at finer spatio-temporal resolutions, making it more feasible to do biodiversity climate research ^10, 11^.

What are the major directions being taken by the field of climate change and biodiversity research in recent years? Are there particular focal topics, or methods, that have drawn most attention? Here we summarise major trends in the recent highly cited literature of this field.

## Filtering and categorising the publications

To select papers, we used the *Web of Science* indexing service maintained by Thomson Reuters, using the term “biodiversity AND (climate change)” to search within article titles, abstracts, and keywords. This revealed 3,691 matching papers spanning the 3-year period 2012 to 2014. Of these, 116 were categorised by *Essential Science Indicators* ( esi.incites.thomsonreuters.com) as being “Highly Cited Papers” (definition: “As of November/December 2014, this highly cited paper received enough citations to place it in the top 1% of [its] academic field based on a highly cited threshold for the field and publication year”), with five also being classed as “Hot Papers” (definition: “Published in the past two years and received enough citations in November/December 2014 to place it in the top 0.1% of papers in [its] academic field”). The two academic fields most commonly associated with these selected papers were “Plant & Animal Science” and “Environment/Ecology”.

Next we ranked each highly cited paper by year, according to its total accumulated citations through to April 1 2015, and then selected the top ten papers from each year (2012, 2013 and 2014) for detailed assessment. We wished to focus on data-oriented research papers, so only those labelled “Article” (Document Type) were considered, with “Review”, “Editorial”, or other non-research papers being excluded from our final list. Systematic reviews that included a formal meta-analysis were, however, included. We then further vetted each potential paper based on a detailed examination of its content, and rejected those articles for which the topics of biodiversity or climate change constituted only a minor component, or where these were only mentioned in passing (despite appearing in the abstract or key words).

The final list of 30 qualifying highly cited papers is shown in Table 1, ordered by year and first author. The full bibliographic details are given, along with a short description of the key message of the research (a subjective summary, based on our interpretation of the paper). Each paper was categorised by methodological type, the aspect of climate change that was the principal focus, the spatial and biodiversity scale of the study units, the realm, biome and taxa under study, the main ecological focus, and the research type and application (the first row of Table 1 lists possible choices that might be allocated within a given categorisation). Note that our choice of categories for the selected papers was unavoidably idiosyncratic, in this case being dictated largely by the most common topics that appeared in the reviewed papers. Other emphases, such as non-temperature-related drivers of global change, evolutionary responses, and so on, might have been more suitable for other bodies of literature. We also did not attempt to undertake any rigorous quantification of effect sizes in reported responses of biodiversity to climate change; such an approach would have required a systematic review and meta-analysis, which was beyond the scope of this overview of highly cited papers.

**Table 1.** Summary information on the 30 most highly cited papers related to climate change effects on biodiversity, for the period 2012–2014. Summary of the ten most highly cited research papers based on the search term: “biodiversity AND (climate change)”, for each of 2012 ^9, 13, 14, 23, 26, 32, 34, 36, 40, 45^, 2013 ^15– 17, 21, 27, 30, 31, 33, 37, 39^ and 2014 ^18– 20, 22, 24, 25, 28, 29, 35, 38^, as determined in the ISI *Web of Science* database. Filters: Reviews, commentaries, and opinion pieces were excluded, as were papers for which climate change was not among the focal topics of the research. The first row of the Table is a key that shows the possible categorisations that were open to selection (more than one description might be selected for a given paper); *n* is the number of times a category term was allocated.

Authors	Year	Title	Journal/Vol/Pg	DOI	Main Message	Type	*n*	Climate Change	*n*	Spatial Scale	*n*	Biodiversity Scale	*n*	Realm	*n*	Biome	*n*	Taxon	*n*	Use	*n*	Ecological Focus	*N*
Author 1 Author 2 Author 3 …then *et al*.	2012 2013 2014	Article title	Publication details Journal, volume Page range	Digital Object Identifier	Key findings of the paper	Methods development Meta-analysis New model Experiment New field data Database Statistical	9 3 5 5 6 14 8	Observed Retrospective validation Reconstruction Future forecast Experimental	9 2 1 19 2	Local Regional Global Multiscale	7 14 7 2	Population Species Community Ecosystem	7 14 8 6	Terrestrial Marine Other	24 8 1	Montane Polar Boreal Temperate Subtropical Tropical Desert Island Riverine Lacustrine Pelagic Benthic Abyssal Global Any	9 3 4 11 6 4 2 0 1 0 3 5 1 4 2	Plant Invertebrate Amphibian Reptile Fish Bird Mammal All	16 4 4 4 4 2 3 5	Theoretical- Fundamental Applied- Management Strategic- Policy	13 17 7	Trait Population dynamics Biogeography Physiology Behaviour Distribution Genetic Migration- dispersal Networks Threatened species Community dynamics Biotic interactions Global change	5 7 3 10 1 16 0 8 1 3 4 2 3
Dullinger, S., Gattringer, A., Thuiller, W., *et al.*	2012	Extinction debt of high- mountain plants under twenty-first- century climate change	Nature Climate Change/ 2/619–622	10.1038/nclimate1514	European Alps plants will suffer average 21stC range contractions of 50% but population dynamics will lag, causing extinction debt	New model, Database		Future forecast		Regional		Community, Species		Terrestrial		Montane		Plant		Strategic-Policy		Population dynamics, Distribution	
Elmendorf, S.C., Henry, G.H.R., Hollister, R.D., *et al.*	2012	Global assessment of experimental climate warming on tundra vegetation: heterogeneity over space and time	Ecology Letters/ 15/164–175	10.1111/j.1461- 0248.2011.01716.x	Response of tundra plants to experimental warming was linear/ cumulative, with no obvious saturating or threshold impacts (indicating lack of feedbacks) but strong regional heterogeneity	Meta-analysis		Experimental		Multiscale		Community, Ecosystem		Terrestrial		Polar, Boreal		Plant		Theoretical- Fundamental		Population dynamics, Community dynamics	
Fordham, D.A., Akçakaya, H.R., Araújo, M.B., *et al.*	2012	Plant extinction risk under climate change: are forecast range shifts alone a good indicator of species vulnerability to global warming?	Global Change Biology/ 18/1357–1371	10.1111/j.1365- 2486.2011.02614.x	It is important to consider direct measures of extinction risk, as well as measures of change in habitat area, when assessing climate change impacts on biodiversity	Methods development, Database		Future forecast		Regional		Species		Terrestrial		Temperate		Plant		Applied- Management		Population dynamics, Distribution, Trait	
Gottfried, M., Pauli, H., Futschik, A., *et al.*	2012	Continent- wide response of mountain vegetation to climate change	Nature Climate Change/ 2/111–115	10.1038/nclimate1329	Based on 60 mountain peaks in Europe plant communities are being transformed by gradual warming, with thermophillic species displacing competitors at a geographically variable pace	Database		Observed		Regional		Community		Terrestrial		Montane		Plant		Theoretical- Fundamental		Trait, Physiology, Community dynamics	
Hickler, T., Vohland, K., Feehan, J., *et al.*	2012	Projecting the future distribution of European potential natural vegetation zones with a generalised, tree species- based dynamic vegetation model	Global Ecology and Biogeography/ 21/50–63	10.1111/j.1466- 8238.2010.00613.x	A new dynamic vegetation model shows that climate change is likely to cause significant shifts in vegetation types in Europe	New model		Future forecast		Regional		Community		Terrestrial		Montane, Boreal, Temperate		Plant		Theoretical- Fundamental, Applied- Management		Biogeography, Distribution	
Mantyka- Pringle, C.S., Martin, T.G., Rhodes, J.R.	2012	Interactions between climate and habitat loss effects on biodiversity: a systematic review and meta-analysis	Global Change Biology/ 18/1239–1252	10.1111/j.1365- 2486.2011.02593.x	In synergy with other threats, maximum temperature was most closely associated with habitat loss, followed by mean precipitation decrease	Meta-analysis, Database		Observed		Global		Population, Community		Terrestrial		Global		All		Strategic-Policy		Global change, Distribution	
Schloss C.A., Nunez, T.A., Lawler, J.J.	2012	Dispersal will limit ability of mammals to track climate change in the Western Hemisphere	Proceedings of the National Academy of Sciences of the United States of America/ 109/8606–8611	10.1073/ pnas.1116791109	Many mammals in the Western Hemisphere will be unable to migrate fast enough to keep pace with climate change	Database, Statistical		Future forecast		Regional - Western Hemisphere		Species		Terrestrial		Montane, Polar, Boreal, Temperate, Subtropical, Tropical, Desert		Mammal		Applied- Management		Distribution, Migration-dispersal	
Sunday J.M., Bates, A.E., Dulvy, N.K.	2012	Thermal tolerance and the global redistribution of animals	Nature Climate Change/ 2/686–690	10.1038/nclimate1539	Thermal tolerance determines the ranges of marine, but not terrestrial, ectotherms	Database, Statistical		Observed		Global		Species		Terrestrial, Marine		Global		Invertebrate, Amphibian, Reptile, Fish		Theoretical- Fundamental, Applied- Management		Biogeography, Physiology, Distribution	
Urban, M.C., Tewksbury, J.J., Sheldon, K.S.	2012	On a collision course: competition and dispersal differences create no-analogue communities and cause extinctions during climate change	Proceedings of the Royal Society B-Biological Sciences/ 279/2072–2080		Interspecific competition and dispersal differences between species will elevate future climate-driven extinctions	Methods development		Future forecast		Local		Community		Terrestrial		Montane		All		Theoretical- Fundamental		Community dynamics, Biotic interactions, Migration-dispersal	
Zhu, K., Woodall, C.W., Clark, J.S.	2012	Failure to migrate: lack of tree range expansion in response to climate change	Global Change Biology/ 18/1042–1052	10.1111/j.1365- 2486.2011.02571.x	Tree species in the US showed a pattern of climate-related contraction in range, or a northwards shift, with <5% expanding. No relationship between climate velocity and rate of seedling spread	Database		Observed		Regional		Population		Terrestrial		Montane, Temperate, Subtropical		Plant		Theoretical- Fundamental		Distribution, Migration-dispersal	
Anderegg, W.R.L., Plavcova, L., Anderegg, L.D., *et al.*	2013	Drought’s legacy: multiyear hydraulic deterioration underlies widespread aspen forest die-off and portends increased future risk	Global Change Biology/ 19/1188–1196	10.1111/gcb.12100	Accumulation of drought- induced hydraulic damage to trees over multiple years leads to increased forest mortality rates and increased vulnerability to extreme events	New field data, Experiment		Observed, Experimental		Local		Population		Terrestrial		Temperate		Plant		Theoretical- Fundamental		Physiology, Population dynamics	
Boetius, A., Albrecht, S., Bakker, K., *et al.*	2013	Export of algal biomass from the melting Arctic sea ice	Science/339/1430–1432	10.1126/ science.1231346	Anomalous melting of summer Arctic sea-ice enhanced the export of algal biomass to the deep-sea, leading to increased sequestering of carbon to oceanic sediments	New field data		Observed		Regional		Ecosystem		Marine		Polar, Pelagic, Benthic		Plant		Theoretical- Fundamental		Global change	
Foden W.B., Butchart, S.H.M., Stuart, S.N., *et al.*	2013	Identifying the World's Most Climate Change Vulnerable Species: A Systematic Trait-Based Assessment of all Birds, Amphibians and Corals	PLoS ONE/8/e65427	10.1371/journal. pone.0065427	Species’ traits associated with heightened sensitivity and low adaptive capacity to climate change can be used to identify the most vulnerable species and regions	Database, Methods development		Future forecast		Global		Species		Terrestrial, Marine		Any		Amphibian, Invertebrate, Bird		Applied- Management, Strategic-Policy		Threatened species, Distribution, Trait	
Franklin, J., David, F.W., Ikeami, M., *et al.*	2013	Modeling plant species distributions under future climates: how fine scale do climate projections need to be?	Global Change Biology/ 19/473–483	10.1111/gcb.12051	The spatial resolution of models influences the location and amount of forecast suitable habitat under climate change	Methods development, Database, Statistical		Future forecast		Regional		Species		Terrestrial		Temperate, Montane		Plant		Applied- Management		Distribution	
Hannah, L., Roehrdanz, P. Ikegami, M., *et al.*	2013	Climate change, wine, and conservation	Proceedings of the National Academy of Sciences of the United States of America/ 110/6907–6912	10.1073/ pnas.1210127110	Climate change will have a substantial impact on suitable habitat for viticulture, potentially causing conservation conflicts	Statistical, Database		Future forecast		Global		Species		Terrestrial		Temperate		Plant		Applied- Management		Distribution	
Harvey B.P., Gwynn-Jones, D., Moore, P.J	2013	Meta-analysis reveals complex marine biological responses to the interactive effects of ocean acidification and warming	Ecology and Evolution/ 3/1016–1030	10.1002/ece3.516	Biological responses of marine organisms are affected by synergisms between ocean acidification and warming	Meta-analysis, Experiment		Future forecast		Multiscale		Population		Marine		Pelagic, Benthic, Abyssal		Plant, Invertebrate, Fish		Theoretical- Fundamental, Applied- Management		Physiology, Population dynamics	
Hazen, E.L., Jorgensen, S., Rykaczewski, R., *et al.*	2013	Predicted habitat shifts of Pacific top predators in a changing climate	Nature Climate Change/ 3/234–238	10.1038/nclimate1686	For a forecast rise of 1–6C in sea-surface temperature, predicts up to a +/-35% change in core habitat of top marine predators	New model, New field data		Future forecast		Regional		Ecosystem		Marine		Temperate, Pelagic		Bird, Fish, Mammal, Reptile		Theoretical- Fundamental, Strategic-Policy		Distribution, Migration-dispersal	
Scheiter, S., Langan, L. Higgins, S.I.	2013	Next- generation dynamic global vegetation models: learning from community ecology	New Phytologist/ 198/957–969	10.1111/nph.12210	Describes features of next- generation dynamic global vegetation models, illustrates how current limits could be addressed by integrating community assembly rules	New model, Methods development		Retrospective validation, Future forecast		Global		Population, Ecosystem		Terrestrial		Boreal, Temperate, Subtropical, Tropical		Plant		Theoretical- Fundamental, Applied- Management		Trait, Physiology, Biogeography	
Smale, D.A., Wernberg, T.	2013	Extreme climatic event drives range contraction of a habitat- forming species	Proceedings of the Royal Society B-Biological Sciences/ 280/20122829	10.1098/ rspb.2012.2829	Extreme warming events can cause population extirpation leading to distribution shifts	New field data, Experiment		Observed		Regional		Species		Marine		Benthic		Plant		Applied- Management		Distribution, Physiology	
Warren, R., VanDerWal, J., Price, J., *et al.*	2013	Quantifying the benefit of early climate change mitigation in avoiding biodiversity loss	Nature Climate Change/ 3/678–682	10.1038/nclimate1887	Analysis of a range of future climate change scenarios shows that over 1/2 plant species and 1/3 mammals likely to lose >50% of range by 2080s; mitigation cuts this substantially	Database, Statistical		Future forecast		Global		Species		Terrestrial		Global		All		Strategic-Policy		Distribution	
Bates, A.E., Barrett, N.S., Stuart-Smith, R.D., *et al.*	2014	Resilience and signatures of tropicalisation in protected reef fish communities	Nature Climate Change/ 4/62–67	10.1038/nclimate2062	Protection from fishing buffers fluctuations in reef fish diversity and provides resistance to climate change	New field data, Statistical		Observed		Local		Community		Marine		Benthic		Fish		Applied- Management		Global change	
Burrows M.T., Schoeman, D.S., Richardson, A.J., *et al.*	2014	Geographical limits to species- range shifts are suggested by climate velocity	Nature/507/492–495	10.1038/nature12976	Global and regional maps of future climate velocity can be used to infer shifts in species distributions	Methods development		Reconstruction, Future forecast		Global		Species		Terrestrial		Global		All		Applied- Management, Strategic-Policy		Migration- dispersal, Distribution	
Hennige, S.J., Wicks, L.C., Kamenos, N.A., *et al.*	2014	Short-term metabolic and growth responses of the cold- water coral *Lophelia* *pertusa* to ocean acidification	Deep-Sea Research Part II-Topical Studies in Oceanography/ 99/27–35	10.1016/ j.dsr2.2013.07.005	Increased levels of atmospheric carbon dioxide will negatively influence the respiration rates, but not calcification rates, of cold- water corals	Experiment		Future forecast		Local		Population		Marine		Benthic		Invertebrate		Theoretical- Fundamental		Physiology	
Jantz, P., Goetz, S., Laporte, N.	2014	Carbon stock corridors to mitigate climate change and promote biodiversity in the tropics	Nature Climate Change/ 4/138–142	10.1038/nclimate2105	If corridors were established to strategically connect tropical forest reserves, would have dual benefit of facilitating dispersal and capturing 15% of currently unprotected carbon stocks	Statistical		Future forecast		Regional		Ecosystem		Terrestrial		Tropical		Plant		Applied- Management		Networks, Migration-dispersal	
Pearson, R.G., Stanton, J.C., Shoemaker, K., *et al.*	2014	Life history and spatial traits predict extinction risk due to climate change	Nature Climate Change/ 4/217–221	10.1038/nclimate2113	Extinction risk from climate change can be predicted using spatial and demographic variables already used in species conservation assessments	Methods development, Database		Future forecast		Regional		Population, Species		Terrestrial		Montane, Temperate, Subtropical, Desert, Riverine		Amphibian, Reptile		Applied- Management		Trait, Population dynamics, Distribution, Migration- dispersal, Threatened species	
Radosavljevic, A., Anderson, R.P.	2014	Making better MAXENT models of species distributions: complexity, overfitting and evaluation	Journal of Biogeography/ 41/629–643	10.1111/jbi.12227	Application of MAXENT to a threatened mouse species to illustrate how species- specific tuning can improve model fit and retrospective validation scores	Statistical, Methods development		Retrospective validation		Regional		Species		Terrestrial		Tropical		Mammal		Theoretical- Fundamental		Distribution, Threatened species	
Scheffers, B.R., Edwards, D.P., Diesmos, A., *et al.*	2014	Microhabitats reduce animal's exposure to climate extremes	Global Change Biology/ 20/495–503	10.1111/gcb.12439	Microhabitats decrease the vulnerability of species and communities to climate change	New field data, Experiment		Future forecast		Local		Species		Terrestrial		Montane		Amphibian, Reptile		Applied- Management		Physiology	
Schmitz, O.J., Barton, B.T.	2014	Climate change effects on behavioral and physiological ecology of predator-prey interactions: Implications for conservation biological control	Biological Control/ 75/87–96	10.1016/ j.biocontrol.2013.10.001	Develops a "habitat domain" framework to help to forecast how climate change will alter predator-prey interactions and biological control	Methods development		Future forecast		Local		Community		Terrestrial		Any		All		Applied- Management		Behaviour, Physiology, Biotic interactions	
Shoo, L.P., O'Mara, J., Perhans, K., *et al.*	2014	Moving beyond the conceptual: specificity in regional climate change adaptation actions for biodiversity in South East Queensland, Australia	Regional Environmental Change/14/435–447	10.1007/s10113-012- 0385-3	Uses case studies from SE Queensland biomes to illustrate the value of context- specific approaches to conservation planning under climate change	Database		Future forecast		Local		Ecosystem		Terrestrial, Other		Subtropical		Plant		Applied- Management		Community dynamics, Physiology	
Zhu, K., Woodall, C.W., Ghosh, S., *et al.*	2014	Dual impacts of climate change: forest migration and turnover through life history	Global Change Biology/ 20/251–264	10.1111/gcb.12382	Tree species in eastern US are not migrating sufficiently to track climate change, and are instead responding with faster turnover rates in warm and wet climates	Database, New model		Observed		Regional		Species		Terrestrial		Temperate, Subtropical		Plant		Strategic- Policy		Migration- dispersal, Population dynamics	

## Analysis of trends, biases and gaps

Based on the categorisation frequencies in Table 1 (counts are given in the *n* columns adjacent to each category), the “archetypal” highly cited paper in biodiversity and climate change research relies on a database of previously collated information, makes an assessment based on future forecasts of shifts in geographical distributions, is regional in scope, emphasises applied-management outcomes, and uses terrestrial plant species in temperate zones as the study unit.

Many papers also introduced new methodological developments, studied montane communities, took a theoretical-fundamental perspective, and considered physiological, population dynamics, and migration-dispersal aspects of ecological change. Plants were by far the dominant taxonomic group under investigation. By contrast, relatively few of the highly cited paper studies used experimental manipulations or network analysis; lake, river, island and marine systems were rarely treated; nor did they focus on behavioural or biotic interactions. Crucially, none of the highly cited papers relied on paleoclimate reconstructions or genetic information, despite the potential value of such data for model validation and contextualisation ^12^. Such data are crucial in providing evidence for species responses to past environmental changes, specifying possible limits of adaptation (rate and extent) and fundamental niches, and testing theories of biogeography and macroecology.

At the time of writing, 5 of the 30 highly cited papers listed in Table 1 (16%) also received article recommendations from *Faculty of 1000* experts ( f1000.com/prime/recommendations) ^9, 13– 16^ with none of the most recent (2014) highly cited papers having yet received an F1000 Prime endorsement.

## Key findings of the highly cited paper collection for 2012–2014

A broad conclusion of the highly cited papers for 2012–2014 (drawn from the “main message” summaries described in Table 1) is that the pace of climate change-forced habitat change, coupled with the increased frequency of extreme events ^15, 17^ and synergisms that arise with other threat drivers ^9, 18^ and physical barriers ^19^, is typically outpacing or constraining the capacity of species, communities, and ecosystems to respond and adapt ^20, 21^. The combination of these factors leads to accumulated physiological stresses ^13, 15, 22^, might have already induced an “extinction debt” in many apparently viable resident populations ^14, 23– 25^, and is leading to changing community compositions as thermophilic species displace their more climate-sensitive competitors ^13, 26^. In addition to atmospheric problems caused by anthropogenic greenhouse-gas emissions, there is mounting interest in the resilience of marine organisms to ocean acidification ^27, 28^ and altered nutrient flows ^16^.

Although models used to underpin the forecasts of climate-driven changes to biotic populations and communities have seen major advances in recent years, as a whole the field still draws from a limited suite of methods, such as ecological niche models, matrix population projections and simple measures of change in metrics of ecological diversity ^7, 12, 29^. However, new work is pushing the field in innovative directions, including a focus on advancements in dynamic habitat-vegetation models ^30– 32^, improved frameworks for projecting shifts in species distributions ^29, 33, 34^ and how this might be influenced by competition or predation ^35, 36^, and analyses that seek to identify ecological traits that can better predict the relative vulnerability of different taxa to climate change ^37, 38^.

In terms of application of the research to conservation and policy, some offer local or region-specific advice on ecosystem management and its integration with other human activities (e.g., agriculture, fisheries) under a changing climate ^18, 24, 35, 39^. However, the majority of the highly cited papers used some form of forecasting to predict the consequences of different climate-mitigation scenarios (or business-as-usual) on biodiversity responses and extinctions ^20– 22, 33, 40^, so as to illustrate the potentially dire consequences of inaction.

## Future directions

The current emphasis on leveraging large databases for evidence of species responses to observed (recent) climate change is likely to wane as existing datasets are scrutinised repeatedly. This suggests to us that future research will be forced to move increasingly towards the logistically more challenging experimental manipulations (laboratory, mesocosm, and field-based). The likelihood of this shift in emphasis is reinforced by the recent trend towards mechanistic models in preference to correlative approaches ^41^. Such approaches arguably offer the greatest potential to yield highly novel insights, especially for predicting and managing the outcomes of future climate-ecosystem interactions that have no contemporary or historical analogue. Along with this work would come an increasing need for systematic reviews and associated meta-analysis, to summarise these individual studies quantitatively and use the body of experiments to test hypotheses.

Technological advances will also drive this field forward. This includes the development of open-source software and function libraries that facilitate and standardise routine tasks like validation and sensitivity analysis of projection or statistical models ^42, 43^, as well as improved access to data layers from large spatio-temporal datasets like ensemble climate forecasts ^10^ and palaeoclimatic hindcasts ^44^. An increasing emphasis on cloud-based storage and use of off-site high-performance parallel computing infrastructure will make it realistic for researchers to undertake computationally intensive tasks ^31^ from their desktop.

These approaches are beginning to emerge, and a few papers on these topics already appear in the highly cited paper list ( Table 1). This includes the innovative exposure of coral populations to varying carbon dioxide concentrations, and the meta-analyses of tundra plant response to experimental warming ^45^ and marine organisms to ocean chemistry ^27^. Such work must also be underpinned by improved models of the underlying mechanisms and dynamic processes, ideally using multi-species frameworks that make use of ensemble forecasting methods for improved incorporation of scenario and climate model uncertainty ^10^. Such an approach can account better for biotic interactions ^41^ *via* individual-based and physiologically explicit “bottom-up” models of adaptive responses ^31^. Lastly, there must be a greater emphasis on using genetic information to integrate eco-evolutionary processes into biodiversity models ^46^, and on improving methods for making the best use of retrospective knowledge from palaeoecological data ^12^.
