# Supplementary material for: Hot topics in biodiversity and climate change research
Source: F1000Res. 2015 Sep 30;4(F1000 Faculty Rev):928. [Version 1] doi: 10.12688/f1000research.6508.1 (PMC4648191; doi:10.12688/f1000research.6508.1)
Supplement: Peer review history

# Peer review: Referee response for version 1

**DOI:** 10.5256/f1000research.6984.r10635

**Published:** 2015-10-01

**Authors:** Bernhard Schmid

**Affiliations:** 1 Institute of Evolutionary Biology and Environmental Studies, University of Zurich, Zurich, CH-8057, Switzerland

I have read this submission. I believe that I have an appropriate level of expertise to confirm that it is of an acceptable scientific standard.

# Peer review: Referee response for version 1

**DOI:** 10.5256/f1000research.6984.r10634

**Published:** 2015-10-01

**Authors:** Jonathan Rhodes

**Affiliations:** 1 Landscape Ecology and Conversation Group, University of Queensland, Brisbane, Qld, Australia

I have read this submission. I believe that I have an appropriate level of expertise to confirm that it is of an acceptable scientific standard.
